# Recent advances in therapies for onychomycosis and its management

**DOI:** 10.12688/f1000research.18646.1

**Published:** 2019-06-25

**Authors:** Aditya K. Gupta, Nadia Stec

**Affiliations:** 1 Division of Dermatology, Department of Medicine, University of Toronto School of Medicine, Toronto, Canada; 2 Mediprobe Research Inc., London, Ontario, Canada

**Keywords:** onychomycosis, tinea pedis, dermatophyte, toenail, antifungal, laser

## Abstract

Onychomycosis is the most common affliction of the nail. It may be caused by dermatophytes, yeasts, and non-dermatophyte molds. Traditionally, oral antifungal treatments have been used to treat the fungus, although they can be accompanied by side effects and drug interactions. Topical treatments provide an alternative modality, bypassing the systemic effects of oral drugs; recent research has centered on topical drug improvement and development. Physical and laser treatments are being used in conjunction with topicals, which may help penetrate the thick nail plate. In this review, techniques from all categories are outlined: both novel experimental approaches and progress and effectiveness of recently developed treatments. More long-term studies are required to determine the efficacy of various treatments, but cure rates are improved when patients adhere to treatments and follow preventative measures to avoid disease recurrence.

## Introduction

Onychomycosis is a fungal infection occurring in the nails and may affect the adjacent skin. Typically, it manifests as discoloration of the nail, nail plate thickening, and onycholysis ^1^. It is the most common nail pathology and accounts for about 90% of toenail infections worldwide ^1^. This infection presents several problems for affected populations, including local pain, paresthesia, and reduced quality of life as its appearance may impair social interactions and daily activities ^2^. Most onychomycoses are caused by the dermatophytes *Trichophyton*, *Trichophyton rubrum*, *Trichophyton mentagrophytes*, and *Epidermophyton* ^3^. Infections often initiate from tinea pedis, a fungal infection on the surrounding skin of the feet ^3^. Factors that contribute to disease progression are humidity, occlusive footwear, nail trauma, and genetic predisposition ^4, 5^. Patients with diabetes, poor peripheral circulation, HIV, and immunosuppression are also more susceptible, as are elderly patients ^6– 10^.

Owing to its chemical composition, the nail is a formidable barrier to the permeation of drugs, and diffusion into the nail is poor relative to the skin ^11^. This, coupled with slow toenail growth, requires that topicals be used for 12 months or longer, ideally until a healthy nail has regrown. Traditionally, oral therapeutics have been the preferred treatment because of their accessibility and efficacy ^2^. Terbinafine and itraconazole are US Food and Drug Administration (FDA)–approved oral antifungal medications, and fluconazole is available as an off-label option in the US. Though effective, they may be accompanied by systemic side effects and drug–drug interactions, which is a concern for those already taking medications for other conditions. Patients may be hesitant to take oral antifungals. It is understandable that there is demand for non-systemic treatments. At present, there are several oral, topical, and physical therapies broadening the treatment options available to patients. Additionally, combination therapy with several drug classes/modalities can be considered ^12^. Some research groups are studying new molecules and permeation enhancers for topicals, diversifying drug targets, and improving the effectiveness of base molecules already in use. When the efficacy of these drugs is measured, commonly used outcome measures are mycological cure and complete cure. Mycological cure is defined as eradication of the fungal pathogen from the nail, confirmed by a negative potassium hydroxide (KOH) preparation (a test to differentiate dermatophytes and yeasts from other skin disorders) and negative fungal culture. Complete cure meets the goals of the patient, physician, and regulatory bodies. Complete cure is defined as 100% clear nail (also known as clinical cure) in addition to mycological cure ^13^. This review will outline some of these recent developments in therapies for managing onychomycosis.

## Building on topical therapies

Topical therapies can effectively treat onychomycosis, particularly when patients adhere to treatment instructions. Owing to the structural nature of the nail, research is focused on improving the delivery of the drug transungually to the nail bed where fungus lies. Unlike their oral counterparts, topical treatments are relatively safe and there is no potential for drug–drug interactions. Properties that influence the permeability of the drug through the nail include molecular weight, lipophilicity, affinity to keratin, ionization, pH, and the ability to sublimate ^14^. It is advantageous to develop a drug delivery system that allows the drug to enter the nail plate through the transungual and subungual routes rather than just penetrating the nail plate, which is demonstrated by the application of tavaborole and efinaconazole ^15^. Phase III clinical trials have been completed for these drugs and further studies are in progress to understand long-term efficacy ^14^. Despite significant advances in the effectiveness of topical treatments, mycological and complete cure rates remain relatively lower than those of some of the oral agents ( Table 1). However, clinical response rates (resulting in cosmetically clear nails) are more favorable.

**Table 1.** List of US Food and Drug Administration–approved drugs and respective cure rates at week 48.

Drug	Mycological cure	Complete cure
Oral medications		
Terbinafine ^20^	70%	38%
Itraconazole ^21^	54%	14%
Topical medications		
Tavaborole ^16^	31.1%	6.5%
Efinaconazole ^22, 23^	53.4–55.2%	15.2–17.8%
Ciclopirox ^24^	29–36%	5.5–8.5%

Tavaborole is a novel boron-based antifungal agent that was approved by the FDA for treating onychomycosis in 2014 ^16^. It penetrates the nail plate because of its small molecular weight and interferes with protein synthesis in fungal cells through its effects on cytoplasmic aminoacyl-tRNA synthetases ^17^. Phase II and III trials have demonstrated its safety and effectiveness for treating mild to moderate onychomycosis (20 to 60% nail involvement). In two phase III randomized, double-blind, vehicle-controlled trials, tavaborole 5% solution was applied once daily for 48 weeks, and efficacy was evaluated at 52 weeks. Patients ranged from 18 to 88 years of age. Mycological cure rates (negative KOH and culture) with tavaborole 5% solution for studies 1 and 2 were 31.1% and 35.9%, respectively, significantly higher than vehicle (7.2% and 12.2%, *P* <0.001). Complete cure rates (100% clear nail and mycological cure) were significantly higher for tavaborole compared with vehicle in study 1 (6.5% and 0.5%, *P* = 0.001) and study 2 (9.1% and 1.5%, *P* <0.001) ^18^. Similarly, a pooled post-study follow-up from these two trials showed that complete cure was higher in the tavaborole-treated group compared with the vehicle control group (28.6% versus 7.7%) at week 60. Additionally, at 60 weeks, mycological cure for the tavaborole group was higher than vehicle (53.1% versus 23.1%) ^19^.

Efinaconazole 10% solution was FDA-approved as a treatment around the same time in 2014. It is a triazole antifungal that inhibits the synthesis of ergosterol in the fungal cell wall. In phase III trials, patients with distal lateral subungual onychomycosis (20 to 50% nail involvement, 18 to 71 years of age) received the solution once daily for 48 weeks and were evaluated at 52 weeks. The results were a 17.8% complete cure rate (0% clinical involvement of nail, negative KOH and culture) versus 3.3% for vehicle in study 1 and 15.2% versus 5.5% in study 2 ( *P* <0.001). Mycological cure rates (negative KOH and culture) were significantly higher with efinaconazole (55.2% in study 1 and 53.4% in study 2) compared with vehicle (16.8% and 16.9%, *P* <0.001) ^12, 22^.

Luliconazole was approved in 2013 for fungal infections of the skin, including tinea pedis in the US ^25^. A 10% cream was investigated as a treatment for onychomycosis. In separate phase IIb/III clinical trials, nail samples were isolated from patients to compare the activity of 10% luliconazole with amorolfine, ciclopirox, and terbinafine against distal subungual onychomycosis ^26^. It showed a mean minimum inhibitory concentration of 0.00022 μg/mL, which was lower than that of the other three antifungals. In a Japanese multicenter, double-blind, randomized phase III study, patients 21 to 79 years of age with 20 to 50% nail involvement were placed into 2:1 groups of once-daily application of luliconazole 5% nail solution and vehicle. After 48 weeks, complete cure (0% clinical involvement of the nail and negative direct microscopy) rate was significantly higher in luliconazole groups (14.9%) compared with vehicle (5.1%, *P* = 0.012). Similarly, the negative direct microscopy rate was significantly higher in luliconazole (45.4%) than vehicle (31.2%, *P* = 0.026). It is suggested that once-daily topical application of luliconazole is clinically effective and well tolerated ^27^. Luliconazole is not approved for the treatment of onychomycosis in the US.

In Europe, ciclopirox 8% hydrolacquer (P-3051) uses a novel technology based on hydroxypropyl chitosan for the delivery of ciclopirox 8% to the nail ^28^. In a randomized, evaluator-blinded, controlled, parallel-group clinical trial, P-3051 showed statistical superiority to amorolfine after 48 weeks in complete cure (negative KOH and culture and no residual clinical involvement of the nail, 35% versus 11.7%, respectively, *P* <0.001) in 120 patients 18 to 75 years of age with 25 to 75% nail involvement. Similarly, mycological cure (negative direct microscopy and culture) was achieved by all patients who received P-3051 compared with 81.7% who received amorolfine ( *P* <0.001) ^29^. In a randomized, evaluator-blinded, placebo-controlled, parallel-group clinical trial comparing P-3051 with reference ciclopirox 8% and placebo, 467 patients (mean age of 49.84 ± 11.89 years) with 25 to 60% nail involvement applied the lacquers for 48 weeks, followed by a 4-week washout period and 8-week follow-up period ^28^. Complete cure (negative KOH microscopy, culture, and 100% growth of a healthy nail at week 48 and washout) was achieved in 5.7% of P-3051 users and 3.2% for reference ( *P* = 0.6834), whereas placebo saw no cure ( *P* = 0.0165). P-3051 complete cure rate increased at 60 weeks (12.7%) and was greater than reference (5.8%, *P* <0.05) and placebo (1.3%, *P* = 0.0029). A post-hoc analysis confirmed that severity of disease is a prognostic factor for responsiveness to P-3051 treatment and significantly affects reported efficacy data ^30^. The population subset excluded patients with more severe disease (>50% nail involvement). P-3051 was superior to placebo and reference ciclopirox in cure and response rates at 60 weeks, and efficacy rates in the P-3051 group were higher in the groups that excluded patients with more than 50% nail involvement. Ciclopirox 8% hydrolacquer (P-3051) is not approved for the treatment of onychomycosis in the US.

A study using polyurethanes (PUs) as new excipients in topical nail treatments has been conducted ^31^. A PU polymer delivered two antifungal drugs (terbinafine and ciclopirox), and a 10% PU concentration was most effective for *in vitro* drug release, permeation, and antifungal activity. The lacquer smooths the nail plate and reduces porosity, increasing effectiveness of the base molecule ^31^. Finally, there are ongoing clinical trials for ME-1111 and MOB-015. ME-1111 is a new agent with potent *in vitro* antifungal activity and small molecular weight ^32, 33^. It targets succinate dehydrogenase of the electron transport chain, inhibiting it and blocking ATP production. MOB-015 is a topical formulation of terbinafine ^34^.

## Oral therapies

Typically, oral therapeutics are reserved for severe infections because of their safety issues and drug–drug interactions ^35^. Recently, there has been insight about the use of ravuconazole and its prodrugs as new drug candidates for oral therapy. A water-soluble prodrug, mono-lysine phosphoester derivative (BFE1224), is in the advanced stages of clinical development ^35^. A phase III randomized, double-blind, placebo-controlled study of fosravuconazole (F-RVCZ) L-lysine ethanolate, the novel oral triazole, was conducted in Japan ^36^. One hundred fifty-three patients 20 to 75 years of age with at least 25% nail involvement were assigned 100 mg of F-RVCZ or placebo to take once daily for 12 weeks. Evaluation was carried out at week 48. The complete cure rates (0% nail involvement and negative KOH) for F-RVCZ and placebo were 59.4% and 5.8%, respectively ( *P* <0.001). Mycological cure rate (negative KOH) was determined every 12 weeks and increased over time; the difference between F-RVCZ and placebo was statistically significant at 24 weeks and onward ( *P* = 0.002 and *P* <0.001 at weeks 36 and 48). At week 48, mycological cure rates were 82.0% for F-RVCZ and 20.0% for placebo ^36^. Fosravuconazole (F-RVCZ) is not approved for the treatment of onychomycosis in the US.

VT-1161 is a novel, tetrazole fungal CYP51 inhibitor designed to selectively target fungal enzymes and maintains high potency for the fungal target ^37^. In a randomized, phase 2b study to evaluate the efficacy and safety of oral VT-1161 for onychomycosis, 259 patients 18 to 70 years of age with 25 to 75% nail involvement at baseline received 300 or 600 mg oral doses or matching placebo. Once-weekly VT-1161 at a dose of 300 or 600 mg was administered for 10 or 22 weeks following a 14-day once-daily loading period at the same dose (300 or 600 mg) ^38^. Mycological cure (negative KOH and culture) at week 48 was shown for 61%to 72% of patients, collapsed across VT-1161 arms, whereas complete cure ranged from 32 to 40% ^38^. Phase II clinical trials have been completed ^39^.

## Physical strategies and laser therapies

Heinlin *et al*. ^40^ showed that repeated, daily cold atmospheric plasma treatment inhibits the *in vitro* growth of *T. rubrum*. Recently, a pilot study demonstrated the effects of non-thermal plasma in treating onychomycosis ^41^. Non-thermal plasma was created by using an electric insulator by short pulses (10 ns) of electric fields that ionize air molecules. This process creates ions, electrons, ozone, hydroxyl radicals, and nitric oxide, which are fungicidal and cytotoxic ^41^. Ultimately, 13 patients 33 to 74 years of age with 25 to 50% nail involvement completed the trial, and 15.4% achieved mycological cure (negative KOH and culture). Additional studies are required because of the small sample size and varying protocols, but it is the first clinical study to report that thermal plasma may be effective against toenail onychomycosis.

Another novel physical strategy is precise laser poration ^42^. Hollow-core photonic crystal fibers guide light from a femtosecond-pulsed laser in a focused, high-energy density beam. As it irradiates the nail, the laser creates pores (100 μm in diameter) with minimal damage to surrounding tissues. Complete poration of nails increases permeation by two to three orders of magnitude relative to an untreated nail, which in turn would increase the effectiveness of topical treatments ^42^. Flores *et al*. ^43^ coupled poration with a nanocapsule formulation of tioconazole. The nano-formulations are advantageous for topical delivery because they ensure stability of actives and act as reservoirs for extended drug delivery. Nanocapsules were composed of pullulan, a water-soluble polysaccharide that forms films ^44^. The newer film-forming formula provided the best efficiency of *ex vivo* delivery, and drug payload percentages were higher than those of marketed products.

Lasers are approved by the FDA because of their similarity to predicate devices. These devices improve cosmetic appearance by increasing the clarity of nail in patients with onychomycosis ^12, 45, 46^. The effectiveness of lasers as a standalone treatment is reported inconsistently, and cure rates for laser treatment are lower than those of oral and topical treatments. There is limited evidence that they can eradicate pathogenic fungi and this is due to incomplete reporting of randomization and lack of controls ^47– 49^. Additionally, the inclusion criteria and definitions of efficacy outcomes between drug and devices differ, preventing meaningful comparisons ^46^. In the US, lasers are approved for the temporary increase of clear nail in onychomycosis ^50^.

Commonly used lasers include neodymium-yttrium garnet lasers and Q-switched laser systems ^1^. The smaller temporal pulse length in this laser is less than the thermal relaxation time of the fungi, which permits contained heating of the fungi in the nail plate while allowing dissipation of heat in the surrounding soft tissue of the toenail and fingernail. There is a lack of robust clinical data. Randomized, double-blind trials are required to determine whether they are actually fungicidal ^51, 52^.

## Adjunctive measures

Aside from adhering to the prescription protocol of their onychomycosis therapy, patients can perform measures to improve the effectiveness of their treatment and avoid the possibility of reinfection. They should disinfect shoes and socks, avoid walking barefoot in public places, keep feet cool and dry, and recognize the early signs of recurrence and reinfection ^4, 53^. It is also important to treat tinea pedis, and any affected family members, early and effectively.

## Conclusions

There is a diverse array of therapies for treating onychomycosis, particularly centered on topical formulas as the adverse effects are limited to the application site without systemic drug interactions. Devices are being considered as an addition to antifungal therapies, which is an important step in diversifying treatment options ^1^.

## Abbreviations

FDA, US Food and Drug Administration; KOH, potassium hydroxide; PU, polyurethane.

## References

[1] Vlahovic TC: Onychomycosis: Evaluation, Treatment Options, Managing Recurrence, and Patient Outcomes. *Clin Podiatr Med Surg.* 2016;33(3):305–18. 10.1016/j.cpm.2016.02.001 27215153

[2] Lipner SR Scher RK: Onychomycosis: Treatment and prevention of recurrence. *J Am Acad Dermatol.* 2019;80(4):853–67. 10.1016/j.jaad.2018.05.1260 29959962

[3] Welsh O Vera-Cabrera L Welsh E: Onychomycosis. *Clin Dermatol.* 2010;28(2):151–9. 10.1016/j.clindermatol.2009.12.006 20347657

[4] Gupta AK Versteeg SG Shear NH: A Practical Guide to Curing Onychomycosis: How to Maximize Cure at the Patient, Organism, Treatment, and Environmental Level. *Am J Clin Dermatol.* 2019;20(1):123–33. 10.1007/s40257-018-0403-4 30456537

[5] Adams C Athanasoula E Lee W: Environmental and Genetic Factors on the Development of Onychomycosis. *J Fungi (Basel).* 2015;1(2):211–6. 10.3390/jof1020211 29376909 PMC5753111

[6] Elewski BE: Onychomycosis: pathogenesis, diagnosis, and management. *Clin Microbiol Rev.* 1998;11(3):415–29. 10.1128/CMR.11.3.415 9665975 PMC88888

[7] Faergemann J Correia O Nowicki R: Genetic predisposition--understanding underlying mechanisms of onychomycosis. *J Eur Acad Dermatol Venereol.* 2005;19 Suppl 1:17–9. 10.1111/j.1468-3083.2005.01283.x 16120201

[8] Gupta AK Konnikov N MacDonald P: Prevalence and epidemiology of toenail onychomycosis in diabetic subjects: a multicentre survey. *Br J Dermatol.* 1998;139(4):665–71. 10.1046/j.1365-2133.1998.02464.x 9892911

[9] Gupta AK Gupta MA Summerbell RC: The epidemiology of onychomycosis: possible role of smoking and peripheral arterial disease. *J Eur Acad Dermatol Venereol.* 2000;14(6):466–9. 10.1046/j.1468-3083.2000.00124.x 11444267

[10] Gupta AK Taborda P Taborda V: Epidemiology and prevalence of onychomycosis in HIV-positive individuals. *Int J Dermatol.* 2000;39(10):746–53. 10.1046/j.1365-4362.2000.00012.x 11095193

[11] Gupchup GV Zatz JL: Structural characteristics and permeability properties of the human nail: A review. *J Cosmet Sci.* 1999;50(6):363–385. Reference Source

[12] Gupta AK Simpson FC: New pharmacotherapy for the treatment of onychomycosis: an update. *Expert Opin Pharmacother.* 2015;16(2):227–36. 10.1517/14656566.2015.993380 25522979

[13] Baran R editor: Baran & Dawber’s diseases of the nails and their management. Fifth edition. Hoboken, NJ: Wiley-Blackwell;2019 Reference Source

[14] Angelo T Borgheti-Cardoso LN Gelfuso GM: Chemical and physical strategies in onychomycosis topical treatment: A review. *Med Mycol.* 2017;55(5):461–475. 10.1093/mmy/myw084 27703019

[15] Gupta AK Simpson FC: Routes of drug delivery into the nail apparatus: Implications for the efficacy of topical nail solutions in onychomycosis. *J Dermatolog Treat.* 2016;27(1):2–4. 10.3109/09546634.2015.1034081 25983025

[16] Anacor Pharmaceuticals Inc: Kerydin (tavaborole) topical solution, 5%.2014; [cited 2016 Mar 8]. Reference Source

[17] Rock FL Mao W Yaremchuk A: An antifungal agent inhibits an aminoacyl-tRNA synthetase by trapping tRNA in the editing site. *Science.* 2007;316(5832):1759–61. 10.1126/science.1142189 17588934

[18] Elewski BE Aly R Baldwin SL: Efficacy and safety of tavaborole topical solution, 5%, a novel boron-based antifungal agent, for the treatment of toenail onychomycosis: Results from 2 randomized phase-III studies. *J Am Acad Dermatol.* 2015;73(1):62–9. 10.1016/j.jaad.2015.04.010 25956661

[19] Gupta AK Hall S Zane LT: Evaluation of the efficacy and safety of tavaborole topical solution, 5%, in the treatment of onychomycosis of the toenail in adults: a pooled analysis of an 8-week, post-study follow-up from two randomized phase 3 studies. *J Dermatolog Treat.* 2018;29(1):44–8. 10.1080/09546634.2017.1329510 28521541

[20] Novartis Pharmaceuticals Canada Inc: Pr LAMISIL* (terbinafine hydrochloride) 250 mg tablets (expressed as base) topical cream 1% w/w (10 mg/g) topical spray solution 1% w/w (10 mg/g) Antifungal Agent. Health Canada Drug Product Database;2013 Reference Source

[21] Janssen Pharmaceutica: SPORANOX® (itraconazole) Capsules.2017 Reference Source

[22] Elewski BE Rich P Pollak R: Efinaconazole 10% solution in the treatment of toenail onychomycosis: Two phase III multicenter, randomized, double-blind studies. *J Am Acad Dermatol.* 2013;68(4):600–8. 10.1016/j.jaad.2012.10.013 23177180

[23] Valeant Canada LP: PrJUBLIATM Efinaconazole Topical Solution, 10% w/w.2013 Reference Source

[24] Aventis Pharma: PENLAC® Nail Lacquer (ciclopirox) Topical Solution, 8%. 01/312004. Reference Source

[25] Valeant Pharmaceuticals Inc: LUZU (luliconazole) Cream, 1% for topical use.2013 Reference Source

[26] Wiederhold NP Fothergill AW McCarthy DI: Luliconazole demonstrates potent *in vitro* activity against dermatophytes recovered from patients with onychomycosis. *Antimicrob Agents Chemother.* 2014;58(6):3553–5. 10.1128/AAC.02706-13 24709260 PMC4068440

[27] Watanabe S Kishida H Okubo A: Efficacy and safety of luliconazole 5% nail solution for the treatment of onychomycosis: A multicenter, double-blind, randomized phase III study. *J Dermatol.* 2017;44(7):753–9. 10.1111/1346-8138.13816 28332720

[28] Baran R Tosti A Hartmane I: An innovative water-soluble biopolymer improves efficacy of ciclopirox nail lacquer in the management of onychomycosis. *J Eur Acad Dermatol Venereol.* 2009;23(7):773–81. 10.1111/j.1468-3083.2009.03164.x 19453778

[29] Iorizzo M Hartmane I Derveniece A: Ciclopirox 8% HPCH Nail Lacquer in the Treatment of Mild-to-Moderate Onychomycosis: A Randomized, Double-Blind Amorolfine Controlled Study Using a Blinded Evaluator. *Skin Appendage Disord.* 2016;1(3):134–40. 10.1159/000441569 27171791 PMC4857848

[30] Piraccini BM Tosti A: Ciclopirox Hydroxypropyl Chitosan: Efficacy in Mild-to-Moderate Onychomycosis. *Skin Appendage Disord.* 2018;5(1):13–9. 10.1159/000488606 30643775 PMC6323372

[31] Valdes BSG Serro AP Marto J: Polyurethanes as New Excipients in Nail Therapeutics. *Pharmaceutics.* 2018;10(4): pii: E276. 10.3390/pharmaceutics10040276 30551686 PMC6321266

[32] Takahata S Kubota N Takei-Masuda N: Mechanism of Action of ME1111, a Novel Antifungal Agent for Topical Treatment of Onychomycosis. *Antimicrob Agents Chemother.* 2016;60(2):873–80. 10.1128/AAC.01790-15 26596944 PMC4750688

[33] Efficacy and Safety Study of ME1111 in Patients With Onychomycosis. ClinicalTrials.gov.2013; [cited 2018 May 16]. Reference Source

[34] Efficacy and Safety of Two Treatment Regimens of Topical MOB015 in Adults With Distal Subungual Onychomycosis. Dept. of Dermatology, Sahlgrenska University Hospital, Gothenburg, Sweden; Report No: NCT01246518.2012 Reference Source

[35] Yamaguchi H: Potential of Ravuconazole and its Prodrugs as the New OralTherapeutics for Onychomycosis. *Med Mycol J.* 2016;57(4):E93–E110. 10.3314/mmj.16-00006 27904057

[36] Watanabe S Tsubouchi I Okubo A: Efficacy and safety of fosravuconazole L-lysine ethanolate, a novel oral triazole antifungal agent, for the treatment of onychomycosis: A multicenter, double-blind, randomized phase III study. *J Dermatol.* 2018;45(10):1151–9. 10.1111/1346-8138.14607 30156314 PMC6220848

[37] Hoekstra WJ Garvey EP Moore WR: Design and optimization of highly-selective fungal CYP51 inhibitors. *Bioorg Med Chem Lett.* 2014;24(15):3455–8. 10.1016/j.bmcl.2014.05.068 24948565

[38] Elewski B Kempers S Bhatia N: Efficacy and Safety of VT-1161 in a Randomized, Double-Blind, Placebo-Controlled Study of Four Oral VT-1161 Regimens in the Treatment of Patients with Moderate-to-Severe Distal-Lateral Subungual Onychomycosis (DLSO). 4th International Summit for Nail Diseases;2017 Reference Source

[39] A Study to Evaluate the Efficacy and Safety of Oral VT-1161 in Patients With Onychomycosis of the Toenail. Report No: NCT02267356.2017 Reference Source

[40] Heinlin J Maisch T Zimmermann JL: Contact-free inactivation of *Trichophyton rubrum* and *Microsporum canis* by cold atmospheric plasma treatment. *Future Microbiol.* 2013;8(9):1097–106. 10.2217/fmb.13.86 24020738

[41] Lipner SR Friedman G Scher RK: Pilot study to evaluate a plasma device for the treatment of onychomycosis. *Clin Exp Dermatol.* 2017;42(3):295–8. 10.1111/ced.12973 28188648

[42] Vanstone S Cordery SF Stone JM: Precise laser poration to control drug delivery into and through human nail. *J Control Release.* 2017;268:72–7. 10.1016/j.jconrel.2017.10.014 29051061

[43] Flores FC Chiu WS Beck RCR: Enhancement of tioconazole ungual delivery: Combining nanocapsule formulation and nail poration approaches. *Int J Pharm.* 2018;535(1–2):237–44. 10.1016/j.ijpharm.2017.11.008 29126904

[44] Flores FC Rosso RS Cruz L: An innovative polysaccharide nanobased nail formulation for improvement of onychomycosis treatment. *Eur J Pharm Sci.* 2017;100:56–63. 10.1016/j.ejps.2016.12.043 28063967

[45] Gupta AK Foley KA Versteeg SG: Lasers for Onychomycosis. *J Cutan Med Surg.* 2017;21(2):114–6. 27815496 10.1177/1203475416677722

[46] Hay R: Therapy of Skin, Hair and Nail Fungal Infections. *J Fungi (Basel).* 2018;4(3): pii: E99. 10.3390/jof4030099 30127244 PMC6162762

[47] Nenoff P Grunewald S Paasch U: Lasertherapie der Onychomykose. *J Dtsch Dermatol Ges.* 2014;12(1):33–8. 10.1111/ddg.12251_suppl 24237592

[48] Nair AB Vaka SR Murthy SN: Transungual delivery of terbinafine by iontophoresis in onychomycotic nails. *Drug Dev Ind Pharm.* 2011;37(10):1253–8. 10.3109/03639045.2011.568946 21457120

[49] Nematollahi AR Badiee P Nournia E: The Efficacy of Ultraviolet Irradiation on *Trichophyton* Species Isolated From Nails. *Jundishapur J Microbiol.* 2015;8(6):e18158. 10.5812/jjm.18158v2 26322201 PMC4548401

[50] Pinpointe USA: 510(k) Summary K093547. PinPointe FootLaser, Pinpointe USA Inc. FDA 510(k) Premarket Notification Database;2010; [cited 2011 Oct 24]. Reference Source

[51] Gupta AK Simpson FC: Laser therapy for onychomycosis. *J Cutan Med Surg.* 2013;17(5):301–7. 10.2310/7750.2012.12060 24067848

[52] Bristow IR: The effectiveness of lasers in the treatment of onychomycosis: a systematic review. *J Foot Ankle Res.* 2014;7:34. 10.1186/1757-1146-7-34 25104974 PMC4124774

[53] Tosti A Elewski BE: Onychomycosis: Practical Approaches to Minimize Relapse and Recurrence. *Skin Appendage Disord.* 2016;2(1– 2):83–7. 10.1159/000448056 27843933 PMC5096127

